# Bronchial hyperreactivity induced by tropomyosins from cockroach and shrimp: a mouse model to study in vivo cross-reactivity

**DOI:** 10.1186/1939-4551-8-S1-A264

**Published:** 2015-04-08

**Authors:** Thalita Martins, Marina Dias, Rafael Prado, Thamires Milani, Adriana S Moreno, Luana Maia, Vânia Bonato, Simone Ramos, Marcos Borges, L Karla Arruda

**Affiliations:** 1 School of Medicine of Ribeirao Preto - University of Sao Paulo, Brazil

## Background

*In vivo* cross-reactivity among tropomyosins, major pan-allergens among invertebrates, is not established. Our aim was to investigate the effects of purified tropomyosins from cockroach (recombinant Per a 7) and shrimp (natural Lit v 1) on airway inflammation and hyperresponsiveness in a mouse model of asthma.

## Methods

Balb/c mice, 4 to 6 weeks-old, were sensitized twice with 50μg of rPer a 7 or nLit v 1 intraperitoneally with 1 mg alum, and challenged with 50μg of rPer a 7 or nLit v 1 intranasally for three days. A group was sensitized with rPer a 7 and challenged with nLit v 1 under same conditions. Controls received saline on same days. Twenty-four hours after the last challenge, mice were ventilated with FlexiVent^®^, and *in vivo* bronchial hyperresponsiveness was evaluated with increased doses of inhaled methacholine (6.25, 12.5, 25 and 50mg/ml). After ventilation, bronchoalveolar lavage fluid (BALF) was collected and cell counts were performed.

## Results

Sensitization and challenge of mice with rPer a 7 or nLit v 1 resulted in increase in bronchial hyperresponsiveness, given by increase in total and tissue resistance and elastance. Total cells in BALF increased in rPer a 7 (1x10^5^ vs 3x10^5^, p<0.01) and nLit v 1 (1x10^5^ vs 1x10^6^, p<0.001) groups, as compared to controls. There was increase in macrophages for rPer a 7 (5x10^4^ vs 1x10^5^, p<0.001) and nLit v 1 (5x10^4^ vs 3x10^5^, p<0.001) and eosinophils for rPer a 7 (2x10^3^ vs 1.4x10^5^, p<0.001) and nLit v 1 (2x10^3^ vs 9.1x10^5^, p<0.001). Mice immunized with rPer a 7 and challenged with nLit v 1 showed no changes in bronchial hyperresponsiveness or eosinophils on BALF as compared to controls (2x10^3^ vs 6x10^3^). However, there was an increase in neutrophils in this group as compared to controls (5x10^4^ vs 1x10^5^, p<0.01).

## Conclusions

Experimental asthma induced by purified tropomyosins from cockroach and shrimp mimicked the main characteristics of human asthma. Despite the high degree of sequence identity and IgE immunologic cross-reactivity, our data suggested that *in vivo* cross-reactivity of these tropomyosins is unlikely.

